# Study of the suitable climate factors and geographical origins traceability of *Panax notoginseng* based on correlation analysis and spectral images combined with machine learning

**DOI:** 10.3389/fpls.2022.1009727

**Published:** 2023-02-07

**Authors:** Chunlu Liu, Zhitian Zuo, Furong Xu, Yuanzhong Wang

**Affiliations:** ^1^ Medicinal Plants Research Institute, Yunnan Academy of Agricultural Sciences, Kunming, Yunnan, China; ^2^ Collge of Traditional Chinese Medicine, Yunnan University of Chinese Medicine, Kunming, Yunnan, China

**Keywords:** *Panax notoginseng*, active components, climate factors, synchronous 2D-COS images, deep learning model, geographical traceability

## Abstract

**Introduction:**

The cultivation and sale of medicinal plants are some of the main ways to meet the increased market demand for plant-based drugs. *Panax notoginseng* is a widely used Chinese medicinal material. The growth and accumulation of bioactive constituents mainly depend on a satisfactory growing environment. Additionally, the occurrence of market fraud means that care should be taken when purchasing.

**Methods:**

In this study, we report the correlation between saponins and climate factors based on high performance liquid chromatography (HPLC), and evaluate the influence of climate factors on the quality of *P. notoginseng*. In addition, the synchronous two-dimensional correlation spectroscopy (2D-COS) images of near infrared (NIR) data combined with the deep learning model were applied to traceability of geographic origins of *P. notoginseng* at two different levels (district and town levels).

**Results:**

The results indicated that the contents of saponins in *P. notoginseng* are negatively related to the annual mean temperature and the temperature annual range. A lower annual mean temperature and temperature annual range are favorable for the content accumulation of saponins. Additionally, high annual precipitation and high humidity are conducive to the content accumulation of Notoginsenoside R1 (NG-R1), Ginsenosides Rg1 (G-Rg1), and Ginsenosides Rb1 (G-Rb1), while Ginsenosides Rd (G-Rd), this is not the case. Regarding geographic origins, classifications at two different levels could be successfully distinguished through synchronous 2D-COS images combined with the residual convolutional neural network (ResNet) model. The model accuracy of the training set, test set, and external validation is achieved at 100%, and the cross-entropy loss function curves are lower. This demonstrated the potential feasibility of the proposed method for *P. notoginseng* geographic origin traceability, even if the distance between sampling points is small.

**Discussion:**

The findings of this study could improve the quality of *P. notoginseng*, provide a reference for cultivating *P. notoginseng* in the future and alleviate the occurrence of market fraud.

## Introduction

Because of the high price of precious Chinese medicinal materials, some criminals often blend pure substances with less expensive materials in order to earn illegal profits (Liu et al., 2019; Ichim and de Boer, 2021). Recently, as a popular medicinal material for the treatment and prevention of diseases and for keeping healthy, *Panax notoginseng* has also been affected by the same situation. A large variety of *P. notoginseng* is sold on the market, which results in some illegal traders mixing the cheap and sub-quality materials with the genuine product, the inferior with the superior (Yao et al., 2021; Yu et al., 2022; Cui et al., 2022). As a common Chinese medicinal material for alleviating blood stasis, hemostasis, swelling, and pain relief, *P. notoginseng* is found in the dried roots of *Panax notoginseng* (Burk) *F. H. Chen* of the Araliaceae family. It is especially suitable for patients with hypertension, hyperlipidemia, hyperglycemia, heart and cerebrovascular diseases, and patients who have low immunity, anemia, and are prone to falling and sprains (Hawthorne et al., 2022; Jiang et al., 2022; Zheng et al., 2022). Additionally, it improves blood circulation, moisturizes the skin, and slows down aging (Peng et al., 2017; Teseo et al., 2021). Phytochemical and pharmacological studies of *P. notoginseng* have demonstrated that its main biologically active components are dammarane-type saponins consisting of protopanaxadiol and protopanaxatriol glycosides (Qiao et al., 2018; Marianela et al., 2021).

The composition of *P. notoginseng* in nature is complex and is highly related to the cultivation years, processing methods, geographical origin, etc. (Wang et al., 2012; Bai et al., 2021; Wan et al., 2021; Zhang et al., 2021). The collection location points of samples are an important factor of geographical origin, which may be related to the content of active components and the market price of *P. notoginseng*. Therefore, it is important to be able to trace the origins of *P. notoginseng*. Climate factors (temperature, light, rainfall) in different geographical collection location points are some of the main factors that cause quality changes in medicinal plants (Liu et al., 2022). Every type of Chinese medicinal material has its own growth preferences, which results in different suitable growth areas. Therefore, it is key to analyze the correlation between the content accumulative of active components and climate factors, and comprehensively assess the influence of climate factors on the quality of *P. notoginseng*. In addition, Yunnan province (especially Wenshan Prefecture) is one of the important growth and export geographic origins of *P. notoginseng*. To prevent confusion about the contents of the *P. notoginseng* that is on the market, it is essential to develop a simple and quick method of geographical origin traceability.

In recent studies, methods have been reported for the traceability of the geographic origins of *P. notoginseng*, such as sensory analysis (macroscopic and microscopic), inductively coupled plasma mass spectrometry (ICP-MS), electronic tongue or electronic nose, and isotope (Tian et al., 2021; Liang et al., 2021; Ji et al., 2022). However, these methods have some disadvantages, such as large variation and subjectivity (sensory analysis), and being expensive, complex, time-consuming, and labor-intensive. Infrared (IR) spectroscopy has the advantages of being rapid, simple, and pollution-free. It has occupied a unique position in the analytical field since its creation, which illustrates its capabilities. With the continuous development of modern technology and the increasing demand for quality detection, IR technology has been widely applied in the research of Chinese medicinal materials (Li et al., 2018; Zhou et al., 2020), food (Wildea et al., 2019; Candoğan et al., 2021), biology (Huber et al., 2021; Kirschbaum et al., 2021), chemistry (Cura et al., 2021; Mishra et al., 2021), and other fields. Among its applications, an IR-based approach to understand the complex composition of *P. notoginseng*, where chemometrics and machine learning models have been developed, has gained great popularity in terms of the possibility of authenticating and tracing the origins of *P. n*o*toginseng*. However, traditional one-dimensional (1D) linear spectra may not be specific enough and can create overlaps of data, which can limit the amount of useful information extracted from data. Being more versatile, two-dimensional correlation spectroscopy (2D-COS) could be used to overcome this drawback and extract useful information from a series of spectra under chemical or physical stimuli (Noda, 1989; Noda, 1990). On the other hand, with the improvement in data processing and analysis, deep learning has become a promising research algorithm for the qualitative detection of Chinese medicinal materials, and it could be used as an auxiliary method for the study of 2D-COS images (Lecun et al., 2015; Jogin et al., 2018). Compared with other methods to trace geographic origins, 2D-COS images combined with the deep learning model do not require complex procedures, such as data processing and feature extraction. 2D-COS is more focused on processing problems of simple digital images, which are easier, faster, and more representative than analyzing complex spectral data itself.

In the past, several reports have studied the traceability of the geographic origins of *P. notoginseng* geographic origins. For example, Bai et al. (2021) generated high performance liquid chromatography (HPLC) characteristic fingerprints of *P. notoginsen*g extract samples by a multi-wavelength fusion profiling (MWFP) method. They used the averagely linear quantified fingerprint method (ALQFM) and an unsupervised statistical method based on fusion fingerprint matching to identify the geographical origins of *P. notoginseng*. Chen et al. (2018) preprocessed through standard normal variables (SNV) and first derivative (FD) for near infrared (NIR) spectra and established a partial least-squares discriminant analysis (PLS-DA) model to quickly identify the geographic origins of *P. notoginseng*. Similarly, Zhou et al. (2020) carried out a single-spectrum analysis and multi-sensor information fusion strategy for Fourier transform mid-infrared (FT-MIR) and NIR data combined with the multivariate classification algorithm to successfully identify the geographic origins of *P. notoginseng*. In contrast, another study used ultraviolet-visible (UV-Vis) spectrophotometry, Fourier transform infrared (FT-IR) spectrum and HPLC combined with chemometrics to determine the total flavonoid content of *P. notoginseng* from different geographic origins. The total flavonoid content was analyzed and predicted by the standard linear equation of rutin and the orthogonal signal corrected partial least squares regression (OSC-PLSR) model, respectively (Li et al., 2017). Meanwhile, some articles have studied the influence of ecological factors on the growth of *P. notoginseng*. For example, He et al. (2016) applied fingerprints of stable oxygen isotope to study the “Dao-di” authenticity of *P. notoginseng* and trace its geographical origins. The dominant ecological factors and their weights affecting the taproot δ^18^O of *P. notoginseng* were studied through correlation analysis, stepwise regression analysis, partial correlation analysis, and path analysis. A total of 16 main ecological factors affecting the taproot δ^18^O of *P. notoginseng* were screened from 49 ecological factors, and the size, direction, decisive factors, restrictive factors, and the dominant factor were analyzed. Additionally, Yue et al. (2022) proposed the theory of *P. notoginseng* regionalization modeling. The ecological suitability of *P. notoginseng* under current and future climates was analyzed by the maximum entropy model (MaxEnt). The study found that the current most suitable habitat for *P. notoginseng* was mainly located in southwest China. Global climate change is not conducive to the development of *P. notoginseng* planting, and climate warming may lead to serious shrinkage of the growth areas of *P. notoginseng*. Considering future climate change, Yunnan Province was still the most suitable habitat area for *P. notoginseng*, and Sichuan Province was an important potential suitable habitat area. The research provided a new perspective on the ecological suitability of other medicinal plants in the southwest mountainous area. Nevertheless, none of these reports were based on HPLC to analyze the correlation between active component content accumulation and climate factors to alleviate the influence of climate factors on the quality of *P. notoginseng*. In addition, there are no reports of using 2D-COS images of NIR data combined with deep learning models to trace the geographic origin of *P. notoginseng* at the levels of district and town.

In this study, to ensure authenticity and traceability, all *P. notoginseng* samples were collected from cultivation bases. HPLC combined with the principal component analysis (PCA) model was used to analyze the differences of *P. notoginseng* between different districts and towns. Correlation analysis and a partial least squares regression (PLSR) model were constructed to research the correlation between the content accumulation of the main components and climate factors of *P. notoginseng* and to analyze the effect of climatic factors on the variation of saponin content under different growth environments. On this basis, in order to prevent the alteration of the product on the market and associated consumer confusion, the geographic origin traceability of *P. notoginseng* from different district levels was further explored by converting raw spectral data into 2D-COS images combined with the ResNet model. In addition, the reliability of the model was verified by identifying the geographic origin of *P. notoginseng* samples from different town levels. The findings of this study could improve the quality of *P. notoginseng*, provide a reference for cultivating *P. notoginseng* in the future, and alleviate the phenomenon of market fraud.

## Materials and methods

### Sample information

As the main objective of the present study was to evaluate the quality of P. notoginseng under the influence of different environmental factors and discrimination the geographical origins. The sampling points were selected from more dispersed locations to be more representative and to allow an analysis of environmental factors. Therefore, the geographical origins were divided into four parts: DDB (Northeastern Yunnan), DDN (Southeast Yunnan), DX (Western Yunnan), and DZ (Central Yunnan). In addition, considering the Yunnan Province, especially Wenshan Prefecture is the main geographical origin of P. notoginseng, it is more meaningful and representative for analysis. Therefore, four town-level samples from Wenshan Prefecture, Yunnan Province were selected for analysis and validation, respectively YS (Yanshan, Wenshan Prefecture), XC (Xichou, Wenshan Prefecture), MG (Maguan, Wenshan Prefecture) and QB (Qiubei, Wenshan Prefecture).

**Figure 1:**
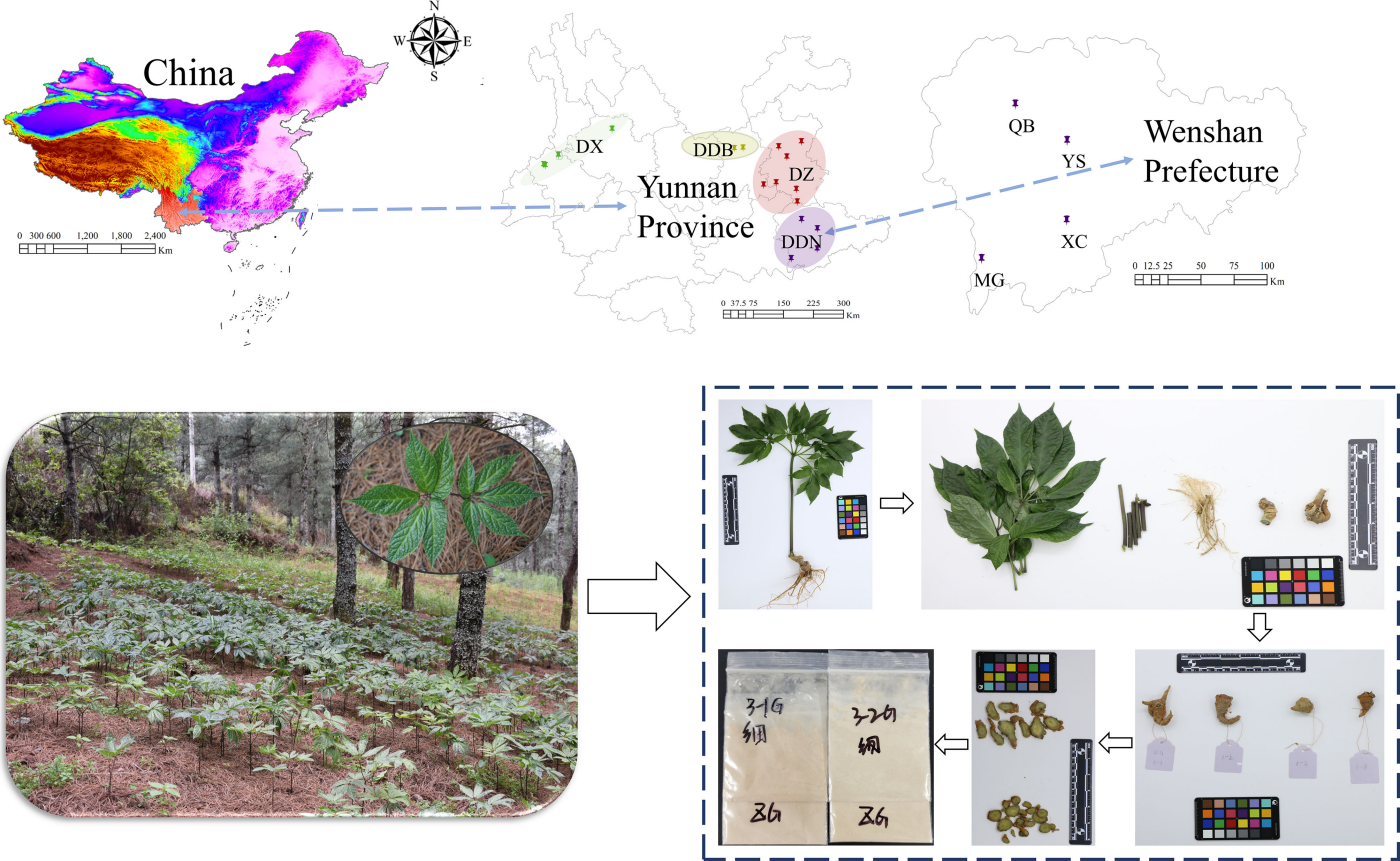
Detailed sample information of the geographical origins, collection locations, and picture of *P. notoginseng*.

A total of 229 *P. notoginseng* samples were collected from the cultivation base of Yunnan province, which meant the authenticity and traceability of the sample could be guaranteed. The altitude ranged from 1150 to 2382 m a.s.l. Detailed sample information of the geographical origins, collection locations, and the corresponding amount have been demonstrated in **Figure 1** and **Table S1**. The collected samples were cleaned with tap water. The different parts were divided and dried at 50°C, then weighed and recorded. Among them, part of the main roots used as the main research object of this research was pulverized and passed through 90 mesh screen. All samples were packaged and labeled in zip-lock bags and stored at room temperature for further use.

### Chemicals reagents

All methanol and acetonitrile used for HPLC analysis were of HPLC grade, and the other chemicals were of analytical grade. Notoginsenoside R1 (NG-R1), Ginsenosides Rg1 (G-Rg1), Ginsenosides Rb1 (G-Rb1), and Ginsenosides Rd (G-Rd) were supplied by the China Institute of Food and Drug Verification (Beijing, China). The UPTL-II-40L system (Chengdu, China) was applied for water purification.

### Climate factors sources

The climate factors (bioclimatic variables and elevation information) were bioclimatic variable layers, which included Bio 1-Bio 19 and a spatial resolution of 30 s. These variables were downloaded from WorldClim (https://www.worldclim.org/), and the detailed information has been shown in **Table S2**. The data (“.tif” format) were opened in ArcGIS 10.6 software, and the climate factor indicators corresponding to the GPS coordinates of *P. notoginseng* at different sampling points were extracted by the “Sampling” tool.

### Reference climate factors and chemical analysis

#### Screening for climate factors

The 19 climate factors may correlate with each other. To avoid co-linearity among these climate factors, a Pearson autocorrelation analysis of the 19 climate factors was conducted by the SPSS 20.0 statistical program. In this study, climate factors that were higher than correlation coefficient (|R|>0.8) and less significant for the distribution of *P. notoginseng* were excluded.

#### Determination of climate factors weights

Principal component analysis (PCA) is an exploratory data analysis technique that uses a smaller number of principal components to represent changes in data sets. The original variables of centered and scaled may be on different measurement scales. After the orthogonal transformation of the normalization, principal components (PCs) were calculated as linear combinations of the original variables. The first PC accounts for more variance and the remaining PC for most likely to occupy the variance not covered by the first PCs. In general, the value of the cumulative variance should be greater than 80% (variance criterion) to be meaningful (Margaritis et al., 2020). PLSR analysis is a multivariate linear regression method that could provide information on the correlation structure of variables and structural similarity or dissimilarity. It can be used to discover correlation models between predictor variables and evaluate the response variables on an equal number of samples. In this study, the variables with more influence in the corresponding models were selected by PLSR (Farrés et al., 2015). The variable importance in projection (VIP) selection method can summarize the effect of each *X* variable on the PLS model and select the variable that contributes most to the explanation of *y* variance. In general, the VIP scores were greater than 1 (the average of the squared), which indicates that the variable makes a significant contribution to the model (Tran et al., 2014). In this study, PCA was used to classify the four saponins in *P. notoginseng*. The linear regression equations of NG-R1, G-Rg1, G-Rb1, G-Rd, and the selected climate factors were established by the PLSR method. Then, according to the linear regression equation, the normal distribution plots of VIP value were obtained. The VIP index value was normalized as the weight coefficient of each climate factor.

### HPLC analysis

The Shimadzu Nexera LC-40 (Kyoto, Japan) device was equipped with an LC-40 binary pump, the SIL-40 automatic sampling device was connected to an SPD-M40 detector, and a Shim-pack VP-ODS column (250 × 4.6 mm, 5 µm) was applied. The mobile phase contained A (water) and B (acetonitrile). The gradient program was as follows: 0-5 min, 20% B; 5-10 min, 20%-25% B; 10-20 min, 25%-28% B; 20-30 min, 28%-30% B; 30-40 min, 30%-36% B; 40-45 min, 36%-40% B; 45-55 min, 40%-45% B; 55-60 min, 45%-90% B; 60-65 min, 90%-20% B; 65-70 min, 20% B. The injection volume for each sample was 10 µL, and the flow rate was 1 mL/min. After each run was balanced (maintain) every 10 min. The column temperature was set at 33-35°C, and the results were monitored at 203 nm. The methodology (linearity ranges, stability, repeatability, precision, and spiked recovery) was investigated by referring to the 2020 edition of the guiding principles of *Chinese Pharmacopoeia* (National Pharmacopoeia Committee, 2020).

### Spectra acquisition

The NIR spectrometer (Thermo Fisher Scientific INC., USA) equipped with a diffused reflection mode was used to measure the spectra of *P. notoginseng*. The sample was placed into a sample cup (confirmed to be radiopaque), and the scanning range was 10000-4000 cm^-1^. The acquisition parameters of each spectrum were scanned 64 times with a resolution of 4 cm^-1^. Each collection was collected twice, and the average spectra were taken for analysis. In addition, it is worth noting that the spectra were corrected by collecting the background to remove atmospheric interference information.

### 2D-COS images acquisition and ResNet model establishment

The 2D-COS is a perturbation-based method first proposed by Noda. In this study, we extend it to generalized 2D-COS image analysis based on 2D spectral theory and literature references (Yang et al., 2013; Yang et al., 2014; Yang et al., 2015; Yang et al., 2020). The variable-variable correlation spectroscopy can set any kind of perturbation variables, such as temperature, concentration, pressure, and time. Synchronized 2D-COS images have sharper characteristic peaks for better characterization of different types of images (Dong et al., 2021). When measuring spectra with equal perturbation intervals *t* in steps *m*, dynamic spectral intensity was represented as a column vector *S* at variable *v*, it was defined as the following:


(1) $$S\left(v\right)=\left[\begin{array}{c} y\left(v,t_{1}\right)\\ y\left(v,t_{2}\right)\\ \begin{array}{c} y\left(v,t_{3}\right)\\ \vdots \\ y\left(v,t_{m}\right) \end{array} \end{array}\right]$$

The synchronous two-dimensional correlation intensities between variables *v_1_* and *v_2_* are calculated as *Φ (v_1_, v_2_)*.


(2) $$\Phi \left(v_{1},v_{2}\right)=\frac{1}{m-1}S{\left(v_{1}\right)}^{T}\cdot S\left(v_{2}\right)$$

**Figure 2:**
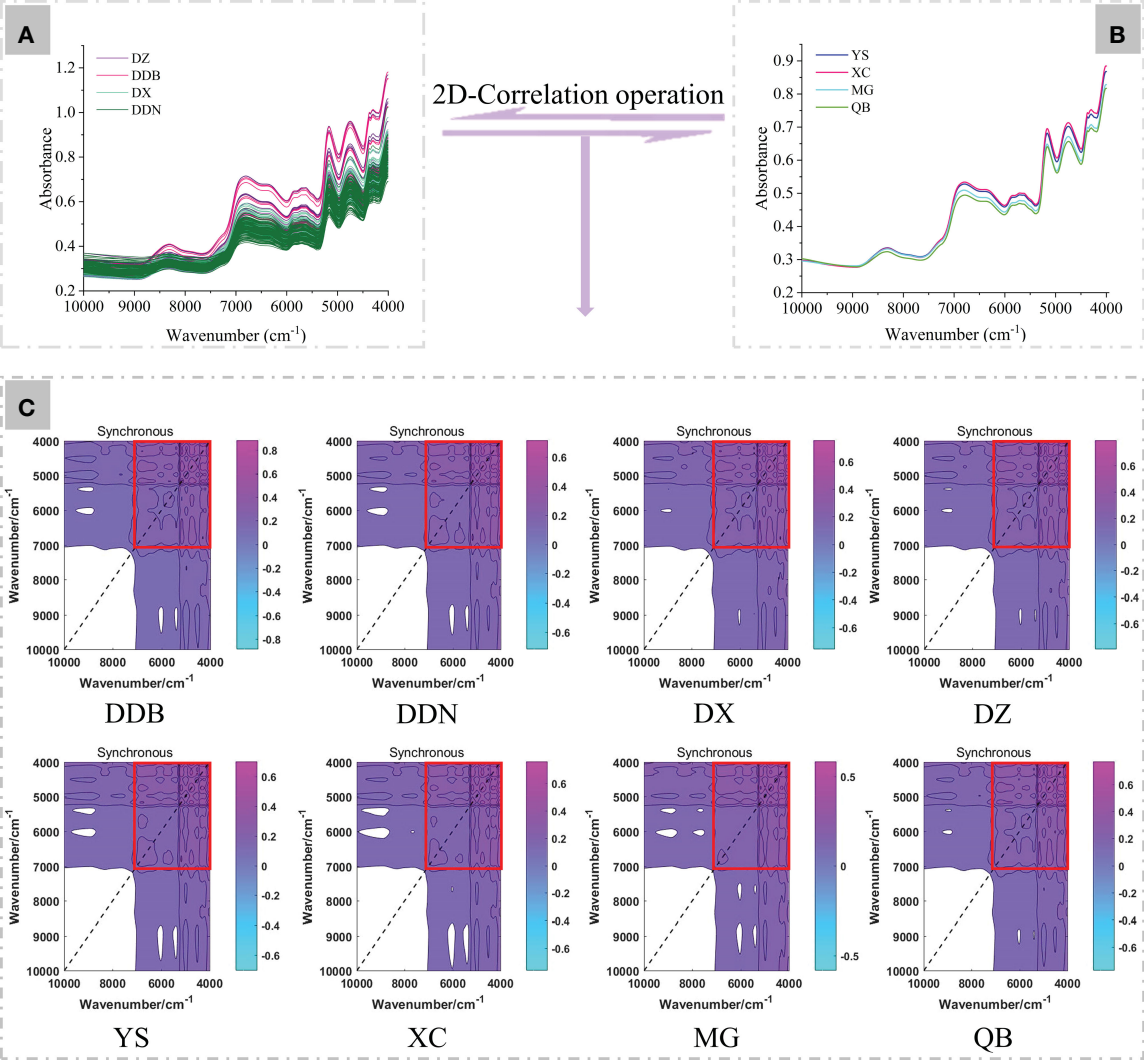
The generation of synchronous 2D-COS **(A, B)**. The original average NIR spectra of *P. notoginseng* from four different districts and towns. **(C)** Full-band synchronous 2D-COS images from different districts and towns. The red box shows the selected synchronous 2D-COS images in the range of 7000-4000 cm^-1^.

According to the full-band 2D-COS images (**Figure 2**), the bands of 7000-4000 cm^-1^ fingerprint area were selected for subsequent analysis. In this study, 90% of the samples (60% as the training set, 30% as the test set) were chosen to establish the ResNet model and the remaining 10% for external validation. Then, a self-built script in MATLAB 2017a was run to generate synchronous 2D-COS images (in the form of JPEG images). This provided a foundation for deep learning modeling. Moreover, we set normalization and resizing in the script to keep the size of images consistent (128×128pixel). We used the MxNet deep learning framework and anaconda3-4.2.0 that comes with Python 3.5.2 to further our learning. Additionally, the TensorBoard and MxBoard were installed for training process visualization and networking.

In this study, the traceability model of *P. notoginseng* from different districts and towns was established by ResNet technology in Convolutional Neural Network (CNN) network. The ResNet technology of deep learning realized residuals with a “shortcut connections” structure, which could simplify learning objectives, reduce training difficulty, speed up the training, and improve the accuracy of the model. The residual module was applied to simplify learning objectives; the detailed process is presented in **Figure S1**. In addition, dimensional consistency of input and output data to determine the structure as identity block or convolution (conv) block was applied. The schematic diagrams of conv and the identity block are shown in **Figure S2A, B**.

**Figure 3:**
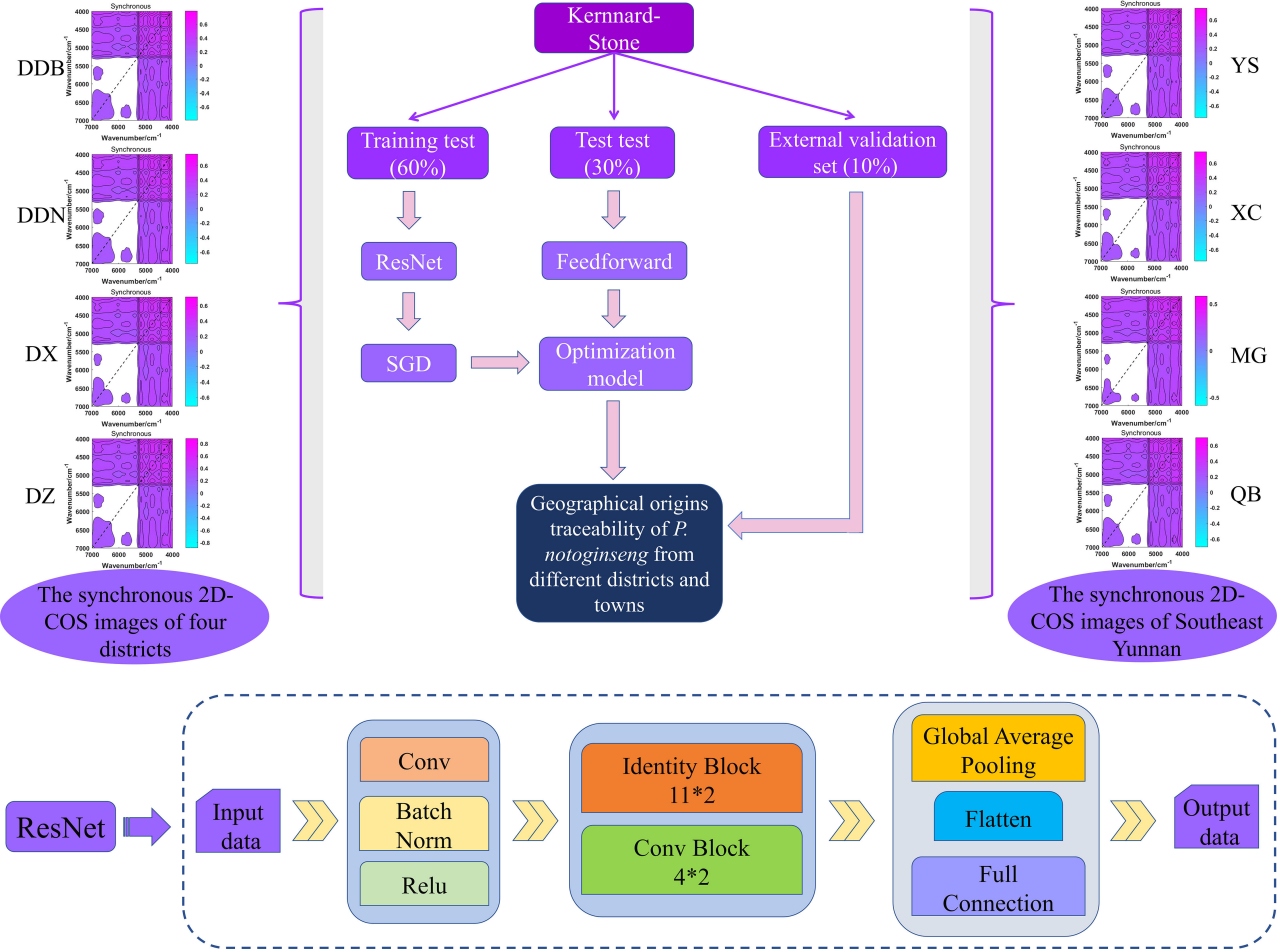
The geographical origins traceability flow chart of *P. notoginseng* from different districts and towns based on ResNet model, and the structure of ResNet model.

The synchronous 2D-COS images acted as the input data. First, a layer of convolution operation is performed on the input data. Then, the BatchNorm normalization and Relu nonlinear activation processing were performed, and the data were input into a 32-layer convolutional neural network (11*2 identity blocks and 4*2 conv blocks) to extract features. The parameters of the fully connected layer were simplified. Additionally, the important features were extracted by global average pooling. Finally, the learned “distributed feature representation” was mapped to the sample label space using the full connection layer output data. The traceability flow chart of geographical origins is shown in **Figure 3**.

## Results and discussion

### Analysis of saponin contents in *P. notoginseng*

**Figure 4:**
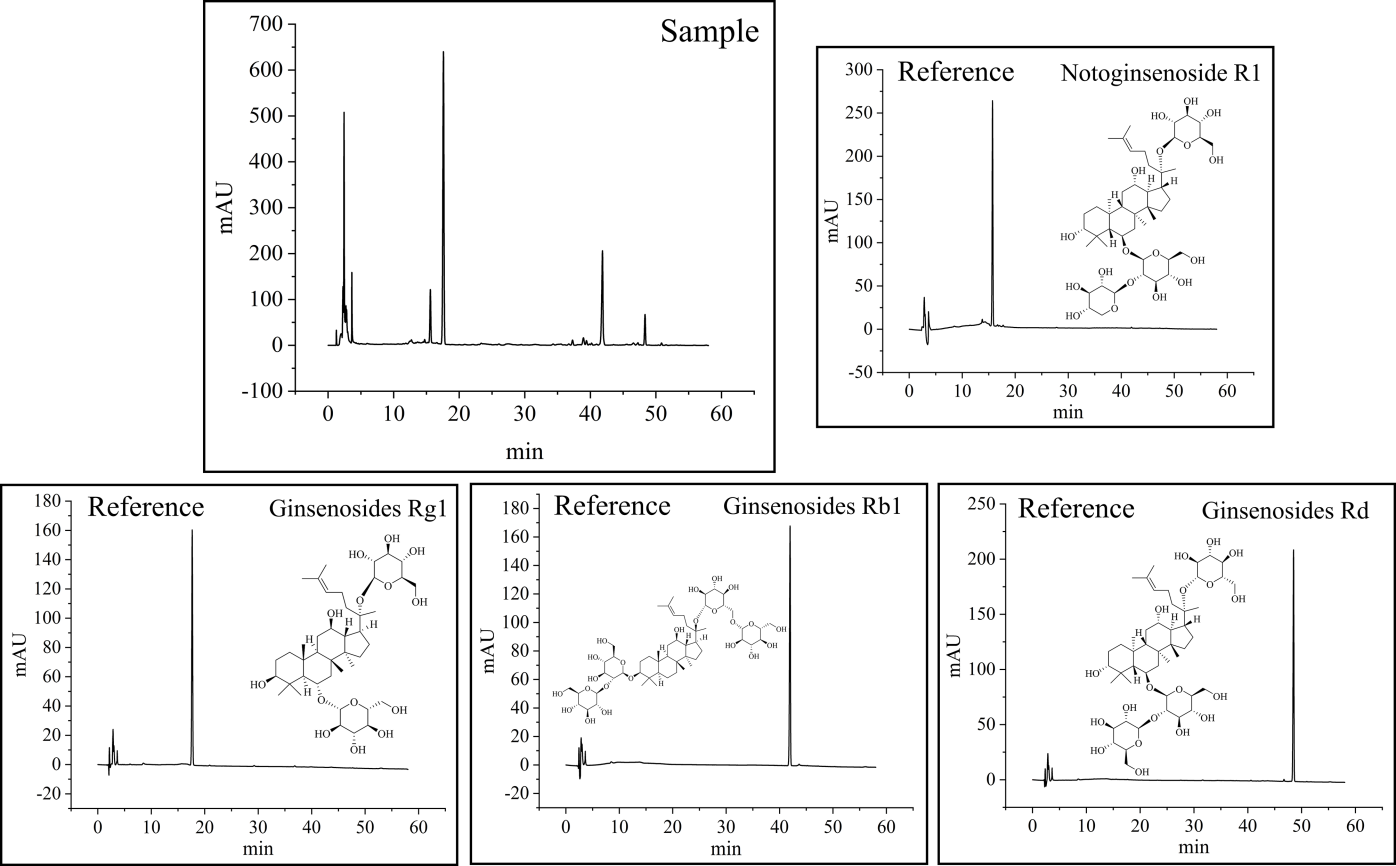
HPLC chromatograms at 203 nm, and the structure of four active compounds in *P. notoginseng*.

*P. notoginseng* is rich in saponins. Among all saponins, NG-R1, G-Rg1, G-Rb1, and G-Rd have the highest content in *P. notoginseng*. They are the most popular compounds applied for quality control of *P. notoginseng* in most studies owing to their excellent biological activity. The HPLC chromatograms and linear regression data of the four saponins are shown in **Figure 4** and **Table S3**. The assay method of HPLC was validated. It is evident from **Table S3** that concentrations and peak areas of the four components show an obvious linear relationship (R^2^>0.9995). The relative standard deviation (RSD) value of stability repeatability, precision, and spiked recovery of each reference compound were all less than 3%. In view of this result, the established method fulfills the requirements for qualitative and quantitative analyses.

According to previous studies, saponins are one of the most important components to exert the drug efficacy of *P. notoginseng*. Saponins are typically used as a quality indicator for evaluating *P. notoginseng*. In this study, combined with *Chinese Pharmacopoeia* (*Chinese Pharmacopoeia* Committee 2020) and literature reports, four saponins in *P. notoginseng* were selected as indicators (Wei et al., 2018; Bai et al., 2021). The level of the four saponins in *P. notoginseng* from different districts and towns is shown in **Table 1**. The results showed that in *P. notoginseng* from different districts and towns, the highest content was of G-Rg1, followed by G-Rb1 and finally G-Rd and NG-R1. This is in line with previous studies by Wei et al. (Wei et al., 2018). In addition, the four saponins in DDB were lower than other districts, and the total average content was 16.55 mg/g. Relatively speaking, the total average content from DDN was the highest at 26.47 mg/g. Among the samples collected from different towns, the total average content from XC was the highest (32.78 mg/g), and the QB provided the lowest (22.04 mg/g). The total average content from YS and MG were similar (27.04 and 26.25 mg/g, respectively). These results indicated that there were certain differences between the content of saponin from different districts and towns. Therefore, comprehensively and properly evaluating the quality of *P. notoginseng* using the four saponins as quality control indicators is feasible. Simultaneously, the above results show the necessity and importance of the identification of *P. notoginseng*. However, there is uncertainty in analyzing the differences of *P. notoginseng* from different districts and towns only based on the content of four saponins. Therefore, the unsupervised multivariate method (PCA) was employed for further analysis.

**Table 1:** Each content and total contents of four main components in *P. notoginseng* from different districts and towns. ( $\bar{x}\pm SD$ ) %.

	Notoginsenoside R1	Ginsenosides Rg1	Ginsenosides Rb1	Ginsenosides Rd	Total (NG-R1+G-Rg1+G-Rb1+G-Rd)
DDB	8.26 ± 1.52	31.28 ± 3.98	19.95 ± 2.47	6.71 ± 1.48	16.55 ± 3.63
DDN	11.39 ± 2.69	44.67 ± 4.04	34.41 ± 3.33	15.44 ± 2.85	26.47 ± 4.21
DX	10.88 ± 2.17	38.68 ± 3.36	32.80 ± 3.57	14.70 ± 2.67	24.26 ± 3.89
DZ	11.01 ± 2.79	36.67 ± 3.60	26.43 ± 3.17	12.01 ± 2.46	21.53 ± 3.75
YS	11.38 ± 2.68	48.07 ± 4.34	35.11 ± 3.22	13.59 ± 3.02	27.04 ± 4.42
XC	15.41 ± 2.98	53.25 ± 4.14	41.83 ± 3.44	20.64 ± 2.94	32.78 ± 4.43
MG	12.90 ± 2.42	46.53 ± 3.54	30.69 ± 3.38	14.87 ± 2.82	26.25 ± 4.09
QB	7.73 ± 2.23	34.52 ± 3.00	31.62 ± 2.86	14.28 ± 2.21	22.04 ± 3.65

### PCA analysis

**Figure 5:**
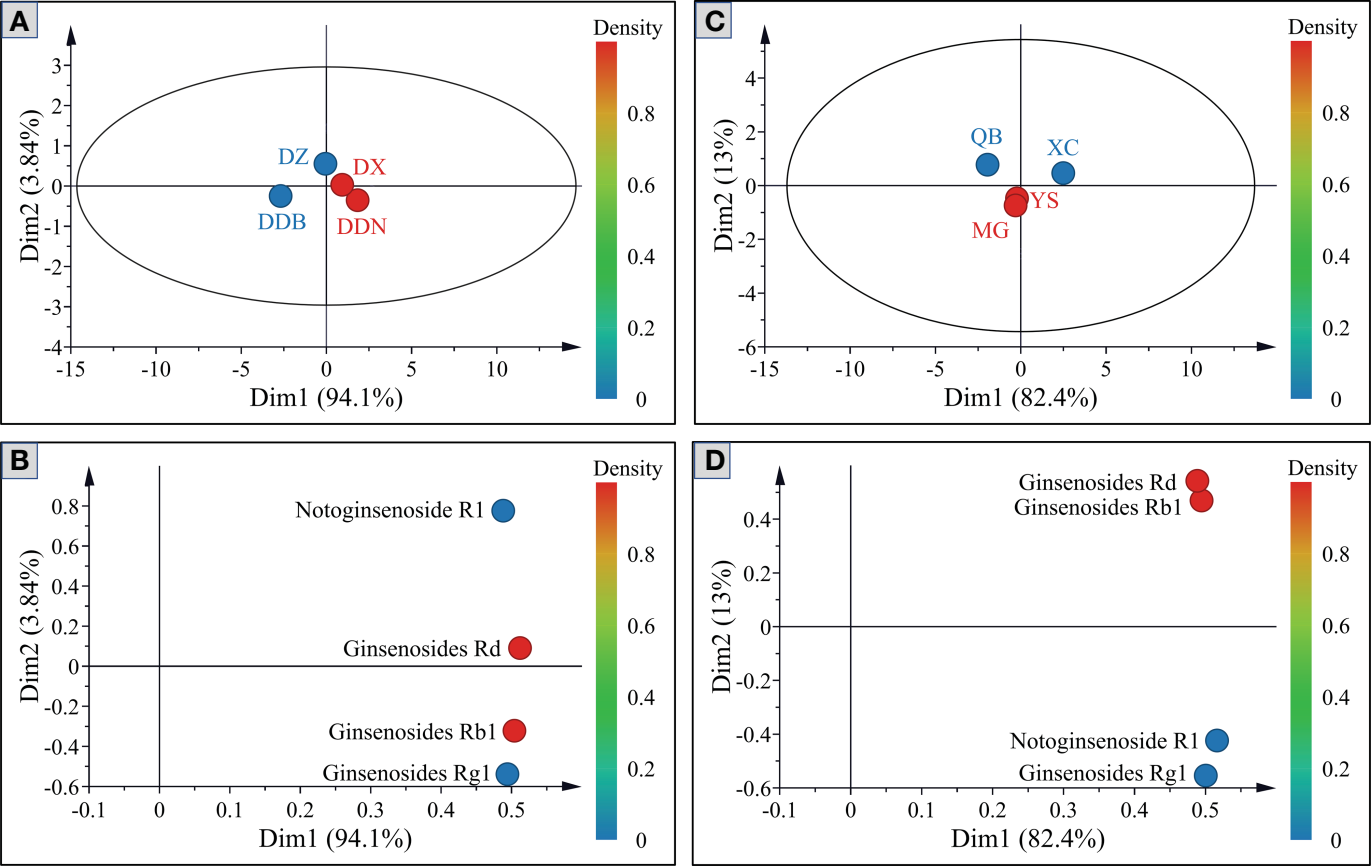
Principal component score plots and loading plots of saponins in *P. notoginseng* from different districts **(A, B)** and towns **(C, D)**.

In order to further reflect the differences of four saponins (NG-R1, G-Rg1, G-Rb1 and G-Rd), PCA was applied to analyze *P.notoginseng* from different districts and towns, respectively. Be seen from **Figures 5A, 5B**, the first two components accounted for 97.94% of the total variance, which could explain most of the information in the sample. From the PCA score plots (**Figure 5A**), *P. notoginseng* from different districts is distributed in different quadrants. The *P. notoginseng* from DDB is located in the fourth quadrant and had a larger dispersion, indicating that saponins contribute substantially to the principal components. Furthermore, they are shown to be negatively correlated with both the first and second principal components. The dispersion of saponins in DZ, DX, and DDN samples is relatively low, which indicated that the component structures of the saponins are relatively similar and could be clustered into one category. However, the contribution rate for the principal components is not high. **Figures 5C, D** present the PCA score plots of *P. notoginseng* from four different towns. The samples of QB and XC have large dispersion, located in the second and third quadrants, respectively. In other words, saponins substantially contribute to the principal components. In addition, there is an overlapping trend between YS and MG, which could be clustered into one category and have a low contribution rate for principal components. The PCA scores scatter plot was established after further analysis. The color of the point represents the contribution of different variables to the principal components. As shown in **Figures 5B, D**, the four saponins varied considerably between different geographic origins. Therefore, the correlation analysis was further carried out, with a view to observing the influence of climate factors on the content accumulation of saponins from different geographic origins.

### Correlation analysis between saponin contents and climate factors

**Figure 6:**
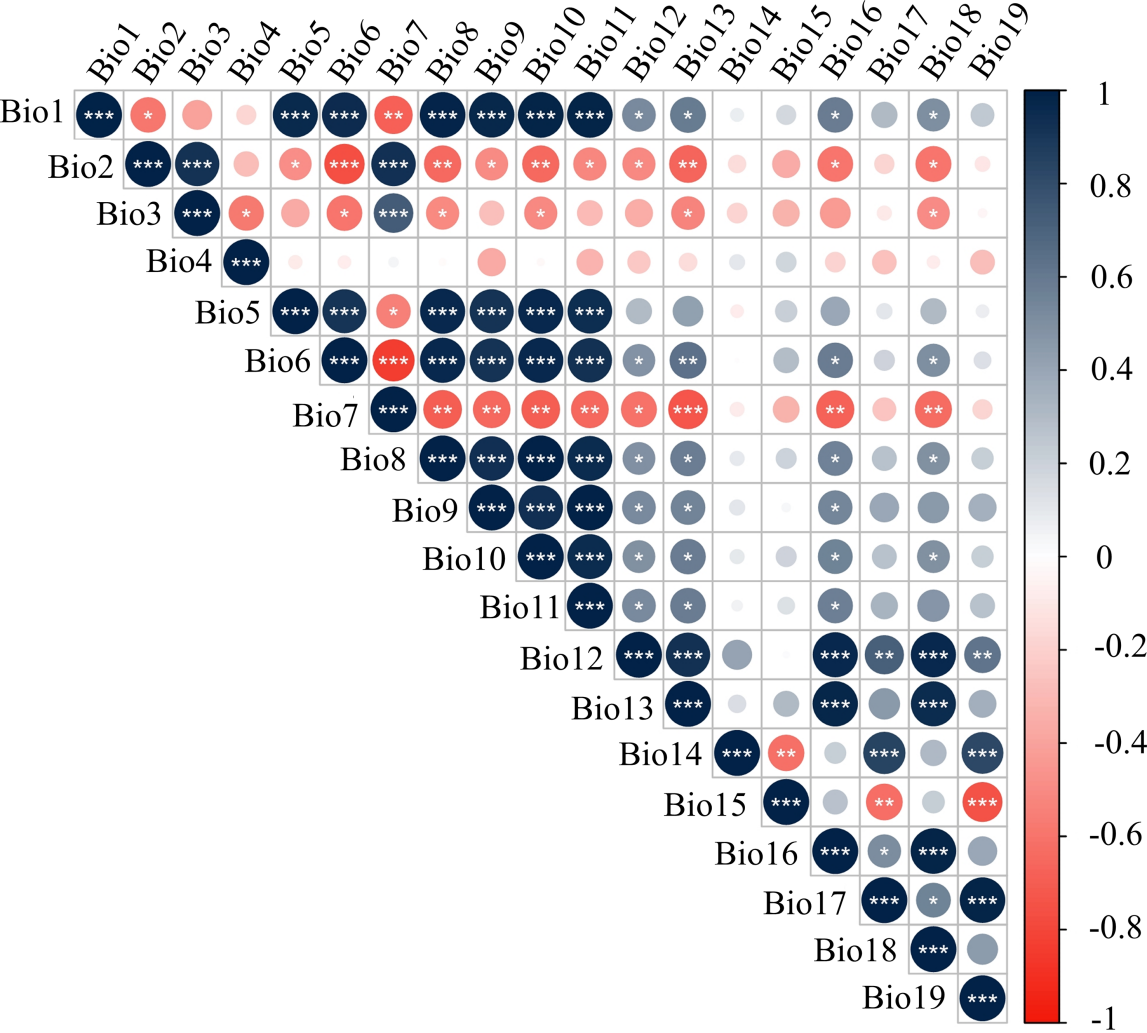
The autocorrelation test of climate factors. The definitions of climate factors are shown in **Table S2**.

Climate factors have a critical effect on the distribution and secondary metabolites of plants. Generally speaking, linear correlation between the independent variables should be examined before constructing a regression model to prevent affecting the fitting effect of the regression model. Therefore, Pearson correlations were used to eliminate climate factors with high correlation coefficients (|R|>8) and less significance for the distribution of *P. notoginseng* samples. The results are shown in **Figure 6**. In the end, a total of seven climate factors (Bio 1, Bio 4, Bio 7, Bio 12, Bio 14, Bio 15, and Bio 17) were obtained for analysis. There was a significant correlation between the level of the four saponins and seven climate factors. Therefore, these seven climate factors were selected as independent variables.

**Figure 7:**
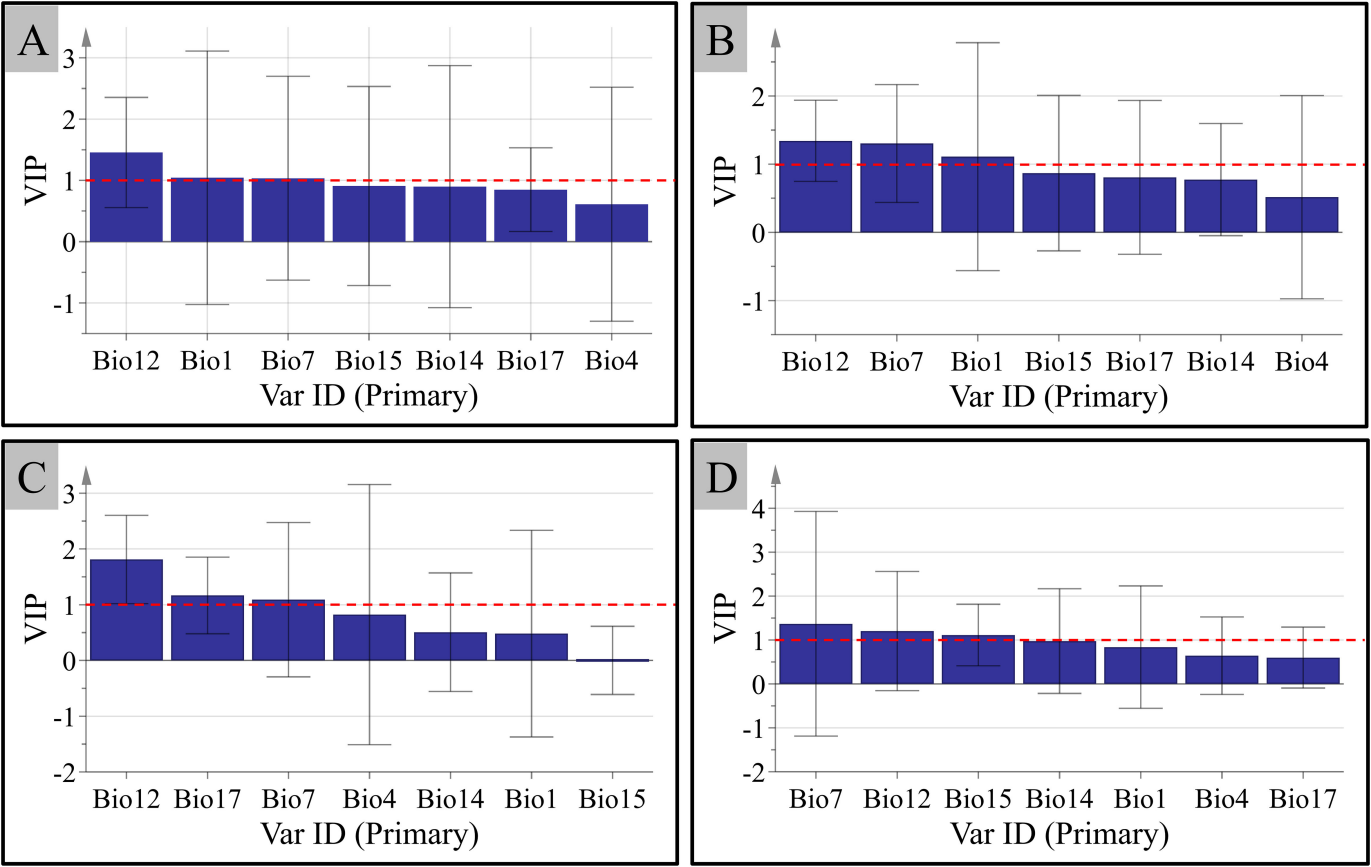
The variable importance in the projection (VIP) between the accumulation of the contents of the four main components in *P. notoginseng* and the climate factors. **(A)** Notoginsenoside R1. **(B)** Ginsenosides Rg1. **(C)** Ginsenosides Rb1. **(D)** Ginsenosides Rd.

The regression equations between the level of the four saponins and seven climate factors established by PLSR are shown below: NG-R1: Y=18.806-0.545 Bio1+0.04 Bio4-0.505 Bio7-0.001 Bio12-0.903 Bio14+0.133 Bio15+0.226 Bio17; G-Rg1: Y=197.720-2.552 Bio1-0.043 Bio4-4.425 Bio7+0.029 Bio12-0.927 Bio14-0.196 Bio15-0.104 Bio17; G-Rb1: Y=110.283-1.221 Bio1-0.046 Bio4-1.185 Bio7+0.037 Bio12-1.144 Bio14-0.406 Bio15-0.141 Bio17, and G-Rd: Y=38.450-0.477 Bio1+0.012 Bio4-0.728 Bio7+0.012 Bio12-1.055 Bio14-0.156 Bio15-0.102 Bio17. The results show that temperature and precipitation are crucial climate factors impacting the content of the four saponins in *P. notoginseng*. The content of NG-R1 correlated negatively with Bio1, Bio7, Bio12, and Bio14, and correlated with Bio4, Bio15, and Bio 17. For G-Rg1 and G-Rb1, their content displayed a negative correlation with Bio 1, Bio 4, Bio 7, Bio 14, Bio 15, and Bio 17, and demonstrated a positive correlation with Bio12. The correlation between the contents of G-Rd and climate factors is similar to G-Rg1 and G-Rb1, the only difference is the positively correlation with Bio4. In the analysis of PLSR, the explanatory power of the independent variable to the dependent variable is measured by the VIP. Therefore, the VIP values of the contents of the four saponins and seven climate factors were analyzed (**Figure 7**). The variables with larger contributions (VIP>1) were screened as important variables. From **Figure 7**, it is clear that Bio7 and Bio12 have a greater impact on the content accumulation of the four saponins. In addition, the content accumulation of the four saponins was also affected by Bio1, Bio1, Bio17, and Bio15, respectively.

To sum up, all the regression coefficients of Bio1 and Bio7 selected based on the VIP value were negative for the four saponins. It shows that these are negatively correlated with the annual mean temperature and the temperature annual range. That is to say, the lower annual mean temperature and the temperature annual range are favorable for the content accumulation of four saponins. DDN (Wenshan Prefecture) is located near the Tropic of Cancer and has a subtropical climate, where the temperature does not experience extremely high or low temperatures. Its annual mean temperature is 15.8°C-19.3°C, and the temperature annual range is small. This may be one of the reasons why Wenshan Prefecture could be regarded as the “Sanqi Hometown”. In addition, the regression coefficients of G-Rg1, G-Rb1, G-Rd, and Bio12 were all positive values, which showed a positive correlation. That is to say, high annual precipitation and high humidity are suitable for the content accumulation of G-Rg1, G-Rb1, and G-Rd. Interestingly, the content accumulation of NG-R1 negatively correlated with Bio12. That is, low annual precipitation could be more suitable for the content accumulation of NG-R1. According to the actual climate analysis of DDN, we speculate that this may be the reason for the low contents of NG-R1 among four saponins. These results are consistent with the traditional production areas of *P. notoginseng*.

### Analysis of original NIR spectra and 2D-COS images

The original average NIR spectra of *P. notoginseng* from four different districts and towns are shown in **Figure 2A, B**. It can be clearly discovered that the spectra at 10000-7600 cm^-1^ have got low signal-to-noise ratios and intensities, which this region probably unsuited for spectra differentiation (Gierlinger et al., 2004). At 7600-5200 cm^-1^ are the first overtone C-H that stretches vibrations in different groups. The peak of 5200-4000 cm^-1^ is the maximal value, which reflects the combined C-H absorption of amino acids, sugars, and proteins (Li et al., 2018; Liu et al., 2019). The broad bands at 8320 cm^-1^ correspond to the second overtone of the C-H stretching in different groups. The bands around 6356 and 6800 cm^-1^ are assigned to the first overtones of the O-H and the N-H stretching. The bands located around 5168 cm^-1^ correspond to the combination of O-H stretching and the first overtone of C-O deformation, and the 4756 cm^-1^ are from the combination of O-H deformation and the C-O stretching. In addition, the absorption band of 4300 cm^-1^ is assigned the combination overtone of C-H and C-C stretching (Nie et al., 2013; Fu et al., 2017; Li et al., 2018; Yang et al., 2019). However, from the original and average NIR spectra, there were less significant differences between the four districts and towns. This may be because the complex composition information of Chinese medicinal materials leads to their similarly existing chemical bonds. Another possible reason would be that the NIR spectra are C-H, O-H, and N-H stretched overtones and combined bands. They are characterized by absorption bandwidths, overlap, and weak absorption, which leads to the characteristics being similar (Nie et al., 2013). As a result, the geographic origins of *P. notoginseng* may be difficult to discriminate directly by the NIR spectra with the naked eye. Therefore, we converted the spectral data into corresponding 2D-COS images combined with the deep learning model for further analysis.

The synchronous 2D-COS images of *P. notoginseng* from different districts and towns are displayed in **Figure 2C**. It can be seen from the synchronous 2D-COS images that the feature peaks are mainly distributed in the 7000-4000 cm^-1^ bands. Therefore, the bands of 7000-4000 cm^-1^ were used for further deep learning modeling.

### Geographical origins traceability analysis of *P. notoginseng* based on ResNet model

The samples of *P. notoginseng* from the four different districts and towns were collected in a relatively large number. The content of *P. notoginseng* from different geographic origins shows great differences due to the influence of climate and human factors. In Wenshan Prefecture, the “Sanqi Hometown, there were also slight differences in the content of samples from different towns. Therefore, tracing geographical origins was carried out of the district level and the town level.

In this study, the weight attenuation coefficient λ of the ResNet model was set to 0.0001, and the learning rate was set to 0.01. In addition, accuracy curves and cross-entropy cost function curves (smoothing parameter is 0.6) of the training set and test set were generated by Mxboard to evaluate the identification ability of the model. The value of accuracy curves is closer to 1, and the cross-entropy cost function is closer to 0, which indicated that the identification ability and convergence effect of the model is better.

**Figure 8:**
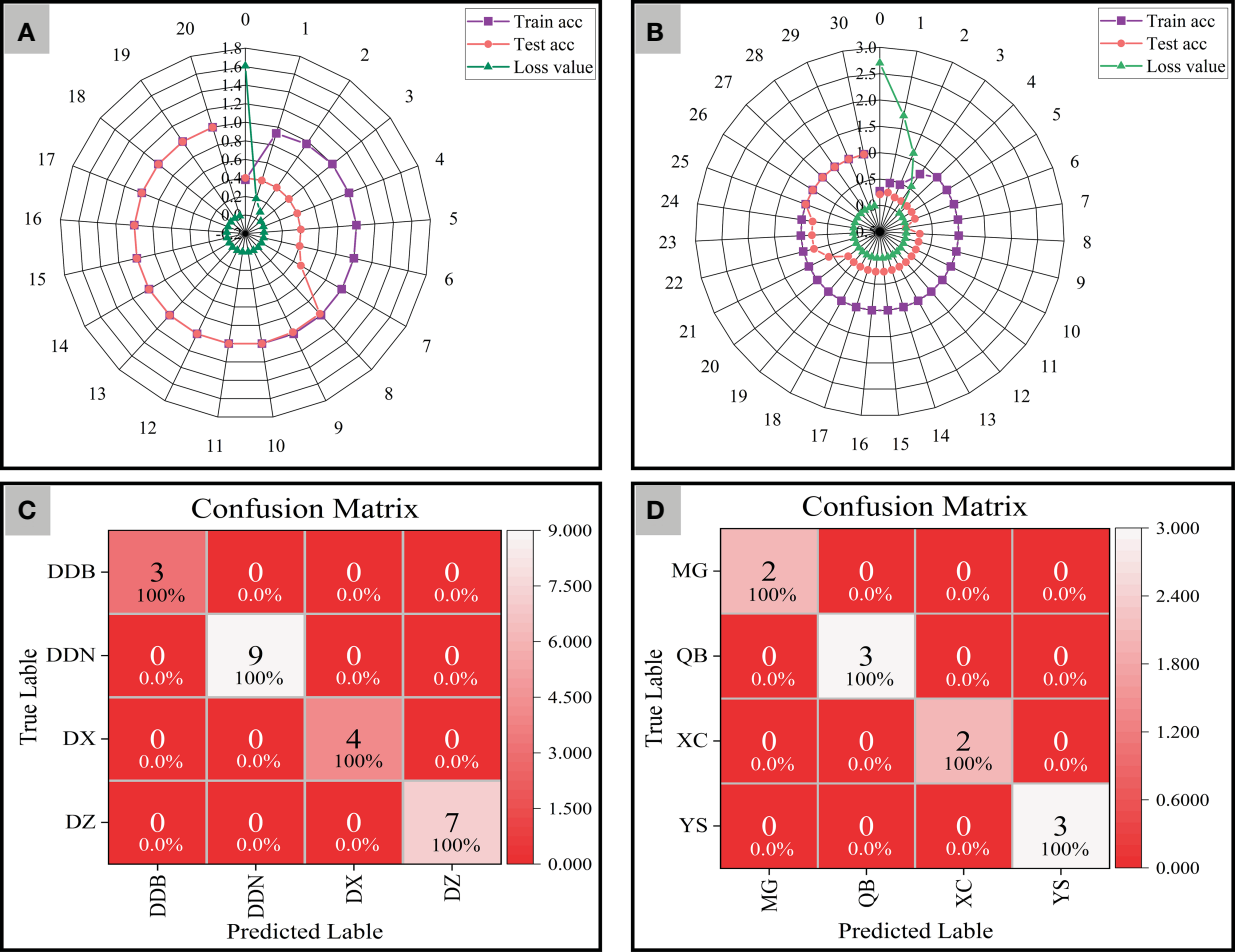
The radar plots of accuracy and cross-entropy cost function of models based on synchronous 2D-COS images (**A**: District; **B**: Town) and the confusion matrix of ResNet models (**C**: District; **D**: Town).

We performed ResNet model analysis on synchronized 2D-COS images of 152 training sets and 54 testing sets from four different districts. The radar plots show the classification accuracy and cross-entropy cost function of the model generated based on synchronized 2D-COS images. As can be seen from **Figure 8A**, the accuracy of both the training set and the test set shows a rising trend. When the number of epochs reaches 10, the accuracy of both the training set and the test set is 1, the loss value is reduced to 0.001, and the model training time is only 675 s. Furthermore, this study applied the established ResNet model for validation on 23 external validation sets, and all external validation samples from four different districts were correctly identified (**Figure 8C**). It shows that the model has strong robustness and could accurately distinguish *P. notoginseng* from different districts in a short time.

As verification, a total of 93 P*. notoginseng* samples were collected from four towns in Wenshan Prefecture and were also analyzed by the same method. As shown in **Figure 8B**, when the number of epochs is 25, the accuracy of both the training set and the test set reaches 1, and the cross-entropy loss value reaches the minimum (0.001). In addition, the results of the confusion matrix demonstrate that the external validation samples are classified correctly (**Figure 8D**). That is to say, the classification models of *P. notoginseng* at different town levels are as good as those at the district level. However, it was not difficult to see that the training time of the model reduced to 597 s.

From the above results, the established model could successfully trace the geographical origins of *P. notoginseng* from different district and town levels. However, the training time of the model may be affected due to the distance between the collected samples. That is to say, the model did not have the phenomenon of overfitting and had strong robustness. The geographical origin of *P. notoginseng* can also be accurately traced when the distance between sampling points is small. The only difference was in the training time of the model, which may be related to the sample number and the differences within the group.

## Conclusion

Some studies from previous literature show that 2D-COS images combined with deep learning can authenticate different herbal and boletus samples, including origin, growth year, and species. In addition, there have been studies that analyzed different bands and different types of 2D-COS images (synchronous, asynchronous, and integrated 2D-COS images). The results of all these studies indicate that synchronous 2D-COS images combined with deep learning is the most suitable method for discrimination analysis. Comparatively, few studies have analyzed climatic factors and quality differences of *P. notoginseng* from different geographical origins .In this study, the method was used to identify the geographical origin. In this study, an identification model of geographical origins of *P. notoginseng* in different districts was proposed and verified by town level samples, the results have indirectly proven the reliability of the model.

Climate is one of the major factors that affects the growth suitability of Chinese medicinal materials, including *P. notoginseng*. Therefore, an investigation into the effects that climate has on the accumulation of active components is essential to improve the quality of *P. notoginseng*. In this study, four saponins of *P. notoginseng* from different districts and towns were determined using HPLC. The correlation between the level of saponins and climate factors was evaluated using PLSR and VIP, and the influence of climate factors on the quality of *P. notoginseng* was analyzed. The results showed that the presence of each saponin was negatively correlated with annual mean temperature and temperature annual range. A lower annual mean temperature and temperature annual range were favorable for the accumulation of the four saponins. In addition, high annual precipitation and high humidity are suitable for the content accumulation of NG-R1, G-Rg1, and G-Rb1, while this is not the case for G-Rd.

In addition, as a traditional Chinese medicinal material with high medicinal value and a high price, *P. notoginseng* is often fraudulently traded on the market. Therefore, a simple and reliable method was proposed to conduct a comprehensive geographic origin traceability study on *P. notoginseng* (from different districts), where the reliability of the model (from different towns) was verified. The results of the accuracy curve, cross-entropy loss function curve, and confusion matrix show that the synchronous 2D-COS model has a strong tendency for generalization. The method proposed in this study could achieve geographical origin traceability of *P. notoginseng*, even though the distance between sampling points is small. The findings of this study could lead to improvements in the quality of *P. notoginseng*, provide a reference for cultivating *P. notoginseng* in the future, and alleviate the phenomenon of market fraud.

## Data availability statement

The raw data supporting the conclusions of this article will be made available by the authors, without undue reservation.

## Author contributions

CL: Data curation and analysis, software, validation, and writing – review and editing. ZZ: validation, project administration, funding acquisition. FX: review – editing, supervision, project administration. YW: Supervision, investigation, resources, project administration. All authors contributed to the article and approved the submitted version.
