# Supplementary material for: Study of the suitable climate factors and geographical origins traceability of Panax notoginseng based on correlation analysis and spectral images combined with machine learning
Source: Front Plant Sci. 2023 Feb 7;13:1009727. doi: 10.3389/fpls.2022.1009727 (PMC9941628; doi:10.3389/fpls.2022.1009727)
Supplement: Supplementary file 1 [file DataSheet_1.docx]

**Study of the suitable climate factors and geographical traceability of *Panax notoginseng* based on correlation analysis and spectral images combined with machine learning**

**Supplementary Figures**

**Supplementary Figure. S1** The schematic diagram of residual block.

**Supplementary Figure. S2** The schematic diagram of conv (A) and identity (B) block.

**Supplementary Tables**

**Supplementary Table S1** The detailed sample information of the geographical origins, collection locations and the corresponding amount of *P. notoginseng* samples.

**Supplementary Table S2** The information of environment variables.

**Supplementary Table S3** The calibration curves and method validation parameters of four main components in *P. notoginseng*.


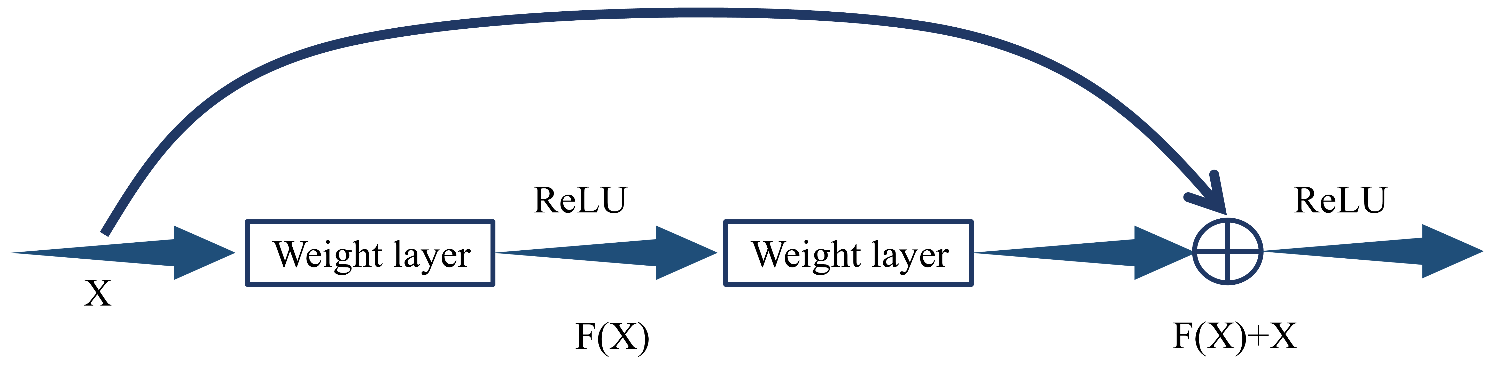


**Figure. S1** The schematic diagram of residual block.


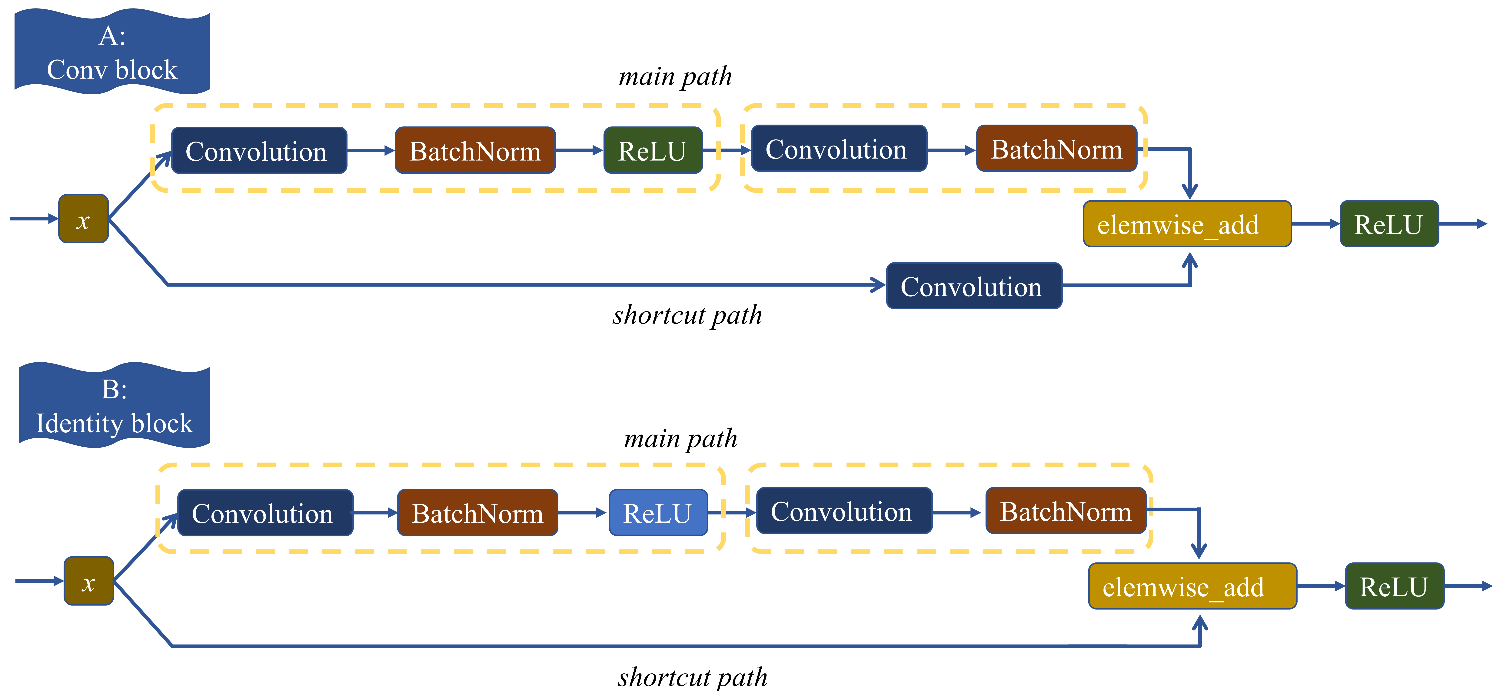


**Figure. S2** The schematic diagram of conv (A) and identity (B) block.

**Table S1** The detailed sample information of the geographical origins, collection locations and the corresponding amount of *P. notoginseng* samples.

| Collection location  origins | Amount | Detail  collection  origins | Amount | Elevation (m) | Latitude | Longitude |
| --- | --- | --- | --- | --- | --- | --- |
| Northeastern Yunnan (DDB) | 27 | Lvquan, Jiulong Town | 18 | 2382 | N 25°48′49″ | E 102°43′13″ |
|  |  | Xundian, Tangdian Town | 9 | 2050 | N 25°49′42″ | E 102°54′44″ |
| Southeast Yunnan (DDN) | 93 | Wenshan, Yanshan County | 27 | 1568 | N 23°47′19″ | E 104°34′01″ |
|  |  | Wenshan, Xichou County | 18 | 1150 | N 23°16′45″ | E 104°33′50″ |
|  |  | Wenshan, Maguan County | 20 | 1722 | N 23°02′01″ | E 103°59′06″ |
|  |  | Wenshan, Qiubei County | 28 | 1451 | N 23°01′17″ | E 104°12′52″ |
| Western Yunnan (DX) | 36 | Dali, Niujie Townships | 9 | 2130 | N 26°18′10″ | E 99°59′51″ |
|  |  | Tengchong, Diantan Town | 9 | 1772 | N 25°38′58″ | E 98°48′13″ |
|  |  | Tengchong, Mingguang Town | 9 | 1990 | N 25°23′05″ | E 98°30′32″ |
|  |  | Tengchong, Diantan Town | 9 | 1807 | N 25°24′18″ | E 98°28′28″ |
| Central Yunnan (DZ) | 73 | Shilin, Tai wan chuang ye yuan | 9 | 1800 | N 24°53′32″ | E 103°22′13″ |
|  |  | Shilin, Changhu Town | 9 | 1895 | N 24°56′57″ | E 103°38′59″ |
|  |  | Xuanwei, Banqiao Town | 18 | 2070 | N 25°59′08″ | E 104°12′49″ |
|  |  | Shizong, Datong Village | 9 | 1944 | N 24°46′56″ | E 104°05′56″ |
|  |  | Shizong, Danuobai Village | 9 | 1993 | N 24°27′57″ | E 104°07′12″ |
|  |  | Qujing, Yanfang Township | 10 | 2184 | N 25°36′02″ | E 103°53′21″ |
|  |  | Qujing, Lingjiao Township | 9 | 1965 | N 25°51′32″ | E 103°42′26″ |

**Table S2** The information of environment variables.

| Abbreviation | Climate factor | Unit |
| --- | --- | --- |
| Bio 1^*^ | Annual mean temperature | ℃ |
| Bio 2 | Mean diurnal range | ℃ |
| Bio 3 | Isothermality | / |
| Bio 4* | Temperature seasonality | ℃ |
| Bio 5 | Max temperature of warmest month | ℃ |
| Bio 6 | Min temperature of coldest month | ℃ |
| Bio 7^*^ | Temperature annual range | ℃ |
| Bio 8 | Mean temperature of wettest quarter | ℃ |
| Bio 9 | Mean temperature of driest quarter | ℃ |
| Bio 10 | Mean temperature of warmest quarter | ℃ |
| Bio 11 | Mean temperature of coldest quarter | ℃ |
| Bio 12^*^ | Annual precipitation | mm |
| Bio 13 | Precipitation of wettest month | mm |
| Bio 14^*^ | Precipitation of driest month | mm |
| Bio 15^*^ | Precipitation seasonality | / |
| Bio 16 | Precipitation of wettest quarter | mm |
| Bio 17^*^ | Precipitation of driest quarter | mm |
| Bio 18 | Precipitation of warmest quarter | mm |
| Bio 19 | Precipitation of coldest quarter | mm |

**Table S3** The calibration curves and method validation parameters of four main components in *P. notoginseng*.

| Compounds | Calibration curves | R^2^ | Linear ranges  (μg/mL) | Stability  RSD (%) | Repeatability  RSD (%) | Precision  RSD (%) | Spiked recovery RSD (%) |
| --- | --- | --- | --- | --- | --- | --- | --- |
| Notoginsenoside R1 | Y=3*10^6^X+16737 | 0.9999 | 8.9-1125.7 | 1.33% | 0.71% | 0.50% | 2.49% |
| Ginsenoside Rg1 | Y=4*10^6^X+20755 | 0.9997 | 20.62-908.2 | 1.18% | 0.22% | 0.23% | 2.80% |
| Ginsenoside Rb1 | Y=3*10^6^X-41956 | 0.9995 | 44.6-1210.9 | 1.11% | 0.19% | 0.21% | 1.30% |
| Ginsenoside Rd | Y=2*10^6^X+40271 | 0.9998 | 11.5-1479.5 | 0.98% | 0.46% | 0.17% | 1.54% |
